# Efficacy of Fish Oil on Serum of TNF*****α*****, IL-1*****β*****, and IL-6 Oxidative Stress Markers in Multiple Sclerosis Treated with Interferon Beta-1b

**DOI:** 10.1155/2013/709493

**Published:** 2013-06-18

**Authors:** V. Ramirez-Ramirez, M. A. Macias-Islas, G. G. Ortiz, F. Pacheco-Moises, E. D. Torres-Sanchez, T. E. Sorto-Gomez, J. A. Cruz-Ramos, G. Orozco-Aviña, A. J. Celis de la Rosa

**Affiliations:** ^1^Laboratorio de Mitocondria-Estrés Oxidativo & Patología, División de Neurociencias, Centro de Investigación Biomédica de Occidente (CIBO), Instituto Mexicano del Seguro Social (IMSS), Sierra Mojada 800, 44340 Guadalajara, JAL, Mexico; ^2^Departamento de Neurología, Unidad Médica de Alta Especialidad (UMAE), Hospital de Especialidades (HE), Centro Médico de Nacional de Occidente (CMNO), IMSS, Guadalajara, JAL, Mexico; ^3^Laboratorio de Bioquímica, Centro Universitario de Ciencias Exactas e Ingenierías, Universidad de Guadalajara, Guadalajara, JAL, Mexico; ^4^Instituto de Investigación de la Inflamación, Guadalajara, JAL, Mexico; ^5^Departamento de Salud Pública, Centro Universitario de Ciencias de la Salud, Universidad de Guadalajara, Guadalajara, JAL, Mexico

## Abstract

Multiple sclerosis (MS) is a chronic inflammatory disease, which leads to focal plaques of demyelination and tissue injury in the central nervous system. Oxidative stress is also thought to promote tissue damage in multiple sclerosis. Current research findings suggest that omega-3 polyunsaturated fatty acids (PUFAs) such as eicosapenta-enoic acid (EPA) and docosahexaenoic acid (DHA) contained in fish oil may have anti-inflammatory, antioxidant, and neuroprotective effects. The aim of the present work was to evaluate the efficacy of fish oil supplementation on serum proinflammatory cytokine levels, oxidative stress markers, and disease progression in MS. 50 patients with relapsing-remitting MS were enrolled. The experimental group received orally 4 g/day of fish oil for 12 months. The primary outcome was serum TNF**α** levels; secondary outcomes were IL-1**β** 1b, IL-6, nitric oxide catabolites, lipoperoxides, progression on the expanded disability status scale (EDSS), and annualized relapses rate (ARR). Fish oil treatment decreased the serum levels of TNF**α**, IL-1**β**, IL-6, and nitric oxide metabolites compared with placebo group (*P* ≤ 0.001). There was no significant difference in serum lipoperoxide levels during the study. No differences in EDSS and ARR were found. *Conclusion.* Fish oil supplementation is highly effective in reducing the levels of cytokines and nitric oxide catabolites in patients with relapsing-remitting MS.

## 1. Introduction

Multiple sclerosis (MS) is a chronic, inflammatory condition of the central nervous system, which affects approximately 2.5 million people worldwide [1]. Nutrition is commonly accepted as one of the possible environmental factors involved in the pathogenesis of MS. Western diet has dramatically decreased the intake of omega-3 essential fatty acids during the last decades [2, 3]. Omega-3 polyunsaturated fatty acids (PUFAs) such as eicosahexanoic acid (EPA) and docosahexaenoic acid (DHA) are fatty acids that possess more than two carbon-carbon double bonds. A diet supplemented with PUFAs has clinical and biochemical effects in patients with autoimmune diseases such as MS [4]. EPA and DHA are found in high proportion in fish oil, and it has been proposed that these molecules may have anti-inflammatory, antithrombotic, antioxidant, and immunomodulatory functions and neuroprotective effects on the synaptogenesis and biogenesis of the neuronal membrane [2, 3, 5–9]. Oxidative stress that is characterized by excessive production of reactive oxygen species and reduction of antioxidant defense mechanisms has been implicated in the pathogenesis of MS [10]. Increased cytokines and oxidative status have been related with the disease progression; therefore, a reduction of proinflammatory cytokines and oxidative stress could be beneficial for MS patients [11]. Interferon beta therapy has been a first-line treatment for relapsing-remitting multiple sclerosis (R-R MS); however, patients continue with inflammation and neurodegeneration [12, 13]. Over the past 60 years, there have been numerous studies on diet and MS, but there is no clear evidence about the efficacy of omega-3 PUFAs as complementary MS treatment [14–17]. The aim of this study was to evaluate the efficacy of fish oil on serum cytokines levels (TNF*α*, IL-1*β*, and IL-6), nitric oxide catabolites, lipoperoxides, expanded disability status scale (EDSS), and annualized relapse rate (ARR) in relapsing-remitting multiple sclerosis.

## 2. Methods 

### 2.1. Participants

This study was a clinical trial on a randomized, double blind placebo-controlled group. Patients were recruited exclusively from the multiple sclerosis clinic of the Neurology Department of the Unidad Médica de Alta Especialidad (UMAE), Hospital de Especialidades (HE), Centro Médico Nacional de Occidente (CMNO), IMSS, Guadalajara, JAL, Mexico. Age of participants was 18–55 years. Patients had clinically definite and magnetic resonance image supported MS, at least one relapse in the year before entry into the study, and a baseline EDSS score of 0–5 and were treated with subcutaneous 250 *μ*g interferon beta-1b (Betaseron, Bayer) every other day at least one year before the trial [18, 19]. 

Patients were excluded if they were taking another supplement; had progressive forms of MS; had history of severe depression; had history of acute liver or renal dysfunction; had history of tobacco, drug, or alcohol abuse; had intolerance, contraindication, or allergy to fish oil; and had customary antioxidant intake. Patients were followed up for at least 1 year. Patients were evaluated at the clinic every 3 months until each patient had reached the 1 year endpoint.

This study was conducted in accordance with the updated Declaration of Helsinki [20] and was approved by the Research Committee of the Social Security Institute of Mexico (Protocol number: R-2010-1301-8). Informed consent was obtained from all patients prior to study enrollment, according to the ethical code of the institution. Identification numbers were assigned to assure patient confidentiality.

### 2.2. Randomization and Blinding

Patients were randomly assigned in a 1 : 1 ratio to receive oral fish oil (4 g/day) or placebo, with a computer-generated randomization sequence (blocks of 2–4). To ensure masking between the fish oil and the placebo, capsules were identical in appearance, packaging, and labeling. Physicians and patients were blind to the intervention. An independent physician evaluated the EDSS score and collected the samples at each clinical visit.

### 2.3. Intervention

Patients received 4 g/day Omega Rx capsules (Dr. Sears zone diet) containing 0.8 g EPA, 1.6 g DHA, and excipient (glycerin, water purified, tocopherol, sunflower oil, and titanium dioxide) or placebo (glycerin, purified water, tocopherol, sunflower oil, and titanium dioxide), orally (4 capsules per day). 

Clinic visits were scheduled every 3 months to assess serum levels of TNF*α*, IL-1*β*, and IL-6, nitric oxide catabolites, lipoperoxides, number of relapses, EDSS, safety, and tolerability. Fasting blood samples were taken at 0, 3, 6, 9, and 12 months. An independent physician assessed the occurrence of side effect at the Neurology Department. In this study, a relapse was defined as new or recurrent neurological abnormalities that were separated by at least 30 days from the onset of the preceding event, lasted at least 24 hours, and occurred without fever or infection.

### 2.4. Outcomes Measurements

Peripheral venous blood (10 mL) was collected into sampling tubes without EDTA. Blood was centrifuged at 3500 rpm for 5 minutes to separate the serum. Serum was stored at –80°C until analysis.

All assays were performed in a blinded fashion on coded samples. Serum levels of TNF*α*, IL-1*β*, IL-6, and NO were measured in duplicate by a sandwich-type enzyme-linked immunosorbent assay (ELISA) technique by using Kits from R&D Systems, TNF alpha DTA00C (range of detection: 15.6–1,000 pg/mL), IL-1b DLB50 (range of detection: 3.9–250 pg/mL), IL-6 D6050, and NO KGE001 (range of detection: 3.12–200 *μ*mol/L). 

A colorimetric assay was used to measure in duplicate the products of lipid peroxidation in serum; a standard curve was run on each assay plate using 1,1,3-trimethoxypropane as standard in serial dilutions. We measured malondialdehyde (MDA) and 4-hydroxyalkenals (HAE) as lipid peroxidation products. The Bioxytech LPO-586 method is designed to assay MDA and HAE using methanesulfonic acid [21].

EDSS progression was measured as a one-point increase sustained for at least 3 months. A relapse was deemed if it was associated with an increase in EDSS by the treating neurologic physician. If any patient had a relapse, the treating physicians indicated intravenous methylprednisolone 1 g/day, for 3 days. We waited one month after the last dose of methylprednisolone for blood sampling. If a patient during the intervention had an infection that required antibiotic treatment, we waited one day after the last dose of antibiotic for blood sampling. The adverse effects of the fish oil treatment were recorded during the clinic visit. A severe adverse effect was defined as any event that causes death and requires hospitalization or prolonged hospital stay.

At study entry and every three months after enrollment, blood samples were collected to ascertain liver function (aspartate aminotransferase, and alanine aminotransferase and alkaline phosphatase); kidney function (urea, creatinine, and uric acid); blood lipids (total cholesterol, high density lipoproteins, low density lipoproteins, and very low density lipoproteins); hemoglobin; leukocytes; platelets; and glucose (data not shown).

The habitual dietary intake, including the essential fatty acids (EPA and DHA), was recorded for all participants. All the patients maintained their diet style and their exercise activity. 

### 2.5. Adhesion and Safety

Participants reported daily consumption of the supplement in a consumption posting sheet. The percentage adherence for each subject was determined by the following formula: (number of pills consumed)/(number of pills returned to the physician) × 100. 

We considered it as an optimal adherence if the percentage was higher than 80%.

### 2.6. Statistical Analysis

For power calculations, we assumed a decrease of 30% serum TNF*α* levels; if at least 21 patients were assigned to each group, this would give a power of more than 80% to detect differences of these magnitudes among groups. We analyzed changes in inflammatory mediators and oxidative stress markers outcomes with respect to each clinic visit with nonparametric and multivariate analysis of variance for repeated measurements (ANOVA) to determine whether there were time differences in each group. Also, we compared the differences between two treatment groups at the same visit with the Mann-Whitney *U* test. Adjustments for multiple comparisons were done with sequential test of Bonferroni. Statistical analyses were done on SPSS version 21 for windows. 

## 3. Results 

**Figure 1:**
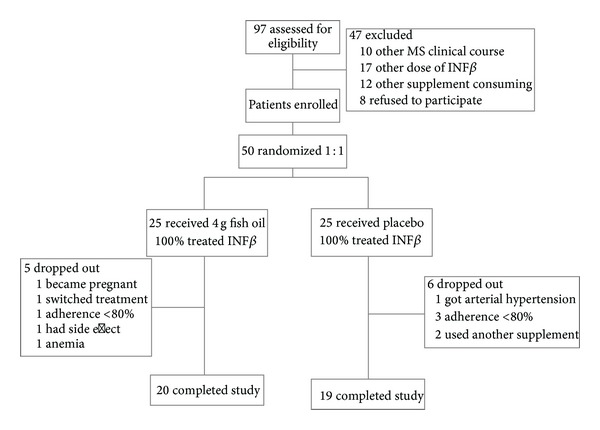
Trial profile.

97 patients were screened, and 50 patients were enrolled and randomly assigned to receive 4 g fish oil (25 patients) or placebo (25 patients). 39 patients completed the trial. One subject in the fish oil group discontinued study participation because of the development of secondary progressive form of multiple sclerosis. 49 patients were treated with interferon beta-1b for at least 12 months.  Figure 1 shows the trial scheme. 

Patients in the two groups had similar demographic and clinical characteristics at baseline (Table 1). In addition, no significant differences in body mass index (BMI), breathing rate, cardiac rate, and blood pressure rate were found during the course of the study. Baseline values of TNF*α*, IL-1*β*, and IL-6, nitric oxide catabolites, lipoperoxides, ARR, and EDSS score were similar in both groups. There was no measurable difference between groups and within subjects in serum levels of lipoperoxides during the study.

### 3.1. TNF*α* Levels

**Figure 2:**
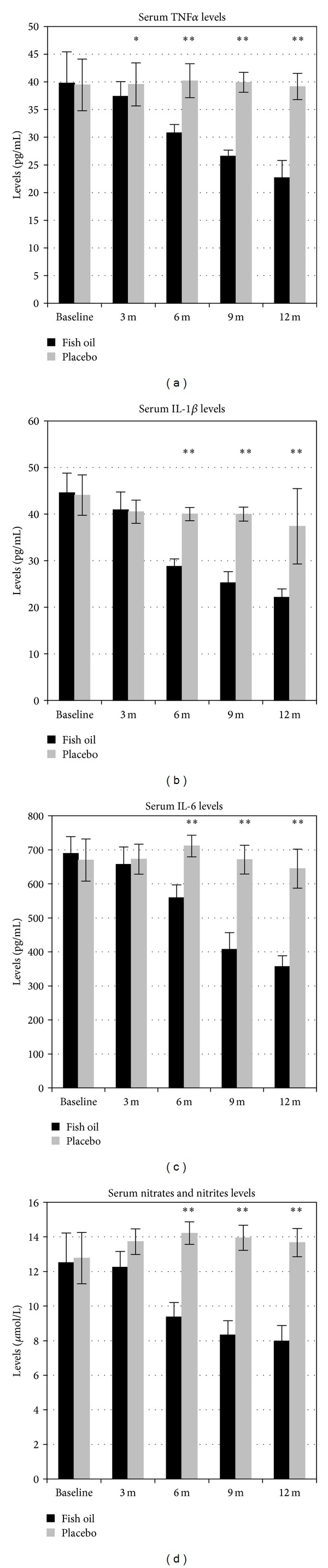
Outcomes at each timepoint in both groups. Data expressed in mean and standard deviation (SD), Mann-Whitney *U* test. Months (m), *P ≤ 0.05, **P ≤ 0.001.

A significant decrease in serum TNF*α* levels was seen at 3, 6, 9, and 12 months for patients in fish oil group, compared with the placebo group (Table 2). After 12 months of fish oil supplementation, there was a decrease of percentage 42.9% compared with 0.7% in placebo.  Figure 2(a) shows the reductions at each time point. Using ANOVA, there was significant change in the differences within subjects in the fish oil group, the difference between 9 months of baseline, 12 months of baseline, and 9 months to 6 months of baseline. TNF*α* levels were different, *P* ≤ 0.001. Within subjects in placebo group show significant differences between 3 months of baseline.

### 3.2. IL-1*β* Levels

A significant decrease in the level of serum IL-1*β* was observed at 6, 9, and 12 months in the fish oil group compared with the placebo group (*P* < 0.001). At 12 months, there was a reduction of serum IL-1*β* level of about 50.3% in the fish oil group, compared with 15.2% in the placebo group (Figure 2(b)). In addition, the serum IL-1*β* levels at 6, 9, and 12 months were significantly reduced in the placebo group compared with the baseline values. 

### 3.3. IL-6 Levels

Serum IL-6 levels decreased at 6, 9, and 12 months, in the fish oil group, compared with placebo (*P* ≤ 0.001). After 12 months, there was a significant reduction in IL-6 levels, and the fish oil decreased to 48.3% compared with 3.8% placebo. There was a significant change in the differences within subjects in the fish oil group, the difference between 3 months of baseline, 6 months of baseline, 9 months of baseline IL-1*β*  levels were different, *P* ≤ 0.001.  Within subjects in placebo group show no significant differences (Figure 2(c)).

### 3.4. Nitric Oxide Catabolites

Nitric oxide catabolites showed a significant decrease after 6 months of fish oil supplementation compared with placebo; this reduction continued until 12 months. The percentage of reduction after 12 months in the fish oil was 36.2% compared with −7% in placebo. The differences within subjects in fish oil group were significant between 3, 6, 9, and 12 months of baseline NO levels, *P* < 0.001. Within subjects in placebo group show no significant differences (Figure 2(d)).

### 3.5. Clinical Outcomes

There were no differences in the EDSS. The mean and standard deviation in fish oil treatment was 2.2 ± 1 points compared to 2.2 ± 0.8 points in placebo. The annualized relapses rate did not change after 12 months in each group; in the fish oil group it was 0.84 ± 0.94 versus placebo group 1 ± 1, *P* = 0.79.

Fish oil supplementation was well tolerated, and there were no serious adverse effects among the groups. No changes were found in the liver and renal test during the 12 months of the trial (data not shown). After 3 months of treatment, the two groups reported fishy taste. Less than 5% of all patients presented nausea, stomach pain, and diarrhea in the first clinic visit (at 3 months). Platelets, blood count, and bleeding did not change during the trial (not shown). Adherence to study treatment was slightly high in the fish oil group; however, this difference was not significant.

## 4. Discussion 

Although it is commonly accepted that nutrition is one of the environmental factors involved in the pathogenesis of MS, its therapy does not involve a particular diet or supplement. In addition, the use of PUFAs as complementary MS treatment is not approved. At present, in R-R MS patients (west of Mexico), there is no evidence about the efficacy of PUFAs contained in fish oil; instead of this, 72% intakes a supplement and ~40% intakes any n-3 PUFAs without medical prescription. The relationship between dietary PUFAs intake and progression of MS remains unclear, but our findings showed anti-inflammatory and antioxidants effects. This evidence is similar to the results of several *in vitro*, *in vivo*, and *postmortem* studies that are relevant to MS [22–26].

This clinical trial showed the efficacy of 4 g fish oil on TNF*α*, IL-1*β*, IL-6, and nitric oxide catabolites levels. Moreover, there were no differences between fish oil and placebo with respect to the lipoperoxides and clinical efficacy outcome (progression on the EDSS and annualized relapse rate). In previous studies, TNF*α*, IL-1*β*, and IL-6 have been implicated as mediators of multiple sclerosis pathology. Increased cytokines have biological actions that lead to axon injury; particularly levels of TNF*α* in cerebrospinal and serum had been associated with disease progression; cerebrospinal fluid levels correlated with the degree of disability (*r* = 0.83, *P* ≤ 0.001) [11]. Bertolotto et al. show an increase of 44% in RNAm levels of TNF*α* in relapsing phases and 22% in remitting phases. PUFAs have anti-inflammatory properties; Caughey et al. reported that 1.62 g/day of EPA and 1.08 g/day of DHA as encapsulated fish oil after 8 weeks inhibited TNF*α* and IL-1*β* synthesis by 70–80%. We observed that 0.8 g/day EPA and 1.6 g/day DHA decreased by 43% in TNF*α*, 50% in IL-1*β*, and 48% in IL-6 levels after 12 months of supplementation [26, 27].

The mechanisms for cytokines decrease by omega-3 fatty acids involve eicosanoid mediators; prostaglandin E_1_ and E_2_ are considered to be inhibitors of TNF*α* and IL-1*β*. The anti-inflammatory effects of EPA and DHA might include competitive inhibition of arachidonic acid, the metabolites of which are involved in promoting inflammation and might also inhibit the migratory activity of leucocytes, via alteration of cytoskeletal components [5, 26, 28–32].

Resolvins and protectins are lipid mediators derived from EPA and DHA via lipoxygenase action [33]. Recently, increasing attention is towards the PUFAs function as ligands for peroxisome proliferator-activated receptors (PPARs) that regulate genes involved in lipid metabolism and anti-inflammatory response; PUFAs might be agonist of PPARs [34, 35]. The inhibition of expression of nuclear factor *κ*b, a transcription factor that is important for the synthesis of inflammatory cytokines and adhesion molecules, has been involved with the intake of omega 3 PUFAs. PUFAs might also reduce the production of matrix metalloproteinases-9 (MMP-9) that have been implicated in disruption of the blood-brain barrier in MS [36]. The initial phase of MS lesion formation is mainly characterized by a cluster of activated microglia and increased cytokines levels without evident signs of demyelination, whereas in the active phase of MS lesion development monocytes invade the lesion and initiate the demyelination process in time, active lesions gradually convert into chronic active lesions, where reactive oxygen species (ROS) and nitric oxide are present. In later stages of MS pathogenesis when inflammation has abates other mechanisms, such as mitochondrial dysfunction, this contributes to the formation of ROS. Normally, local antioxidant enzymes counteract the amount of ROS produced; in MS, ROS production is markedly increased, and the local antioxidant is not responding. In our study, there were no significant differences in the lipid peroxidation products, and we only observed a significant decrease in products of nitric oxide by 36.2% after 12 months in the fish oil group [37].

In addition, TNF*α*, IL-1*β* and INF*γ* can induce another cytotoxic effector molecule, inducible nitric oxide synthase (iNOS). This enzyme is present in actively demyelinating lesions, and stable reaction product of nitric oxide such as nitrates and nitrites is increased in the CSF and serum of patients with MS [38–42]. Several studies have suggested a role for nitric oxide and its oxidizing molecules, such as peroxynitrite in the immunopathogenesis of MS. In this study, we found that PUFAs contained in fish oil have antioxidant function since this decreases the nitric oxide products, and this reduction could be beneficial for MS patients [43]. 

Previously, Swank studied PUFAs intake contribution to risk and progressions in MS; however, their study had some deficiencies. Our study tried to control some confounding outcomes; there were no changes in the daily diet intake and physical exercise [14–17]. However, in this study, no differences in clinical outcomes were found. In others studies there was evidence about the efficacy on EDSS and the relapses number; MS patients (*n* = 12) who intaked EPA and DHA for 4 months showed minimal change in disability; only 5/12 of patients had R-R disease; they decreased 3.30 to 2.7 of EDSS score [44]. Another study with 47 patients showed decrease in the frequency and severity of exacerbations, mostly after the first year of observation. In a similar trial, 16 patients of R-R MS received DHA and EPA dose for 2 years, but the investigators also reduced the daily saturated fat intake and increased weekly fish consumption; after 2 years the patient showed a significant reduction in the annual relapse rate and EDSS score (*P* ≤ 0.001) [45]. It is important to remark that those studies and their methodology could affect the results. In our trial, there was a minimal decrease in the number of relapses in the fish oil group, but this reduction was not a significant difference [14–17]. Recently, Torkildsen et al. in a clinical trial double-blinded, randomized, and multicentered study showed in 46 MS patients that 1.3 g/day EPA and 0.85 g/day DHA during 2 years had no significant difference on the annual relapse rate and EDSS score. Omega-3 PUFAs supplementation given as monotherapy or in combination with interferon beta had no effect on the progression outcome. We used similar doses of EPA (0.8 g/day) and DHA (1.6 g/day), and we also did not find change in the clinical outcomes [6]. However, in this trial the primary outcome was not to evaluate the efficacy on the clinical outcome, and the primary outcome was on the TNF*α* levels; this could be that the sample size did not have sufficient power to detect small and medium treatment effects on ARR and EDSS. There were limitations in this study; one was the sample size to evaluate the clinical outcomes, and the other was that we did not evaluate the severity on each relapse and the time between each one.

## 5. Conclusions 

We show efficacy of 4 g/day fish oil, orally, for the reduction of TNF*α*, IL-1*β*, IL-6, and NO levels compared with placebo. Therefore, 4 g fish oil seems to have efficacy to decrease inflammatory cytokines and nitric oxide catabolites in relapsing-remitting multiple sclerosis. However, no differences in clinical efficacy were seen after 12 months of supplementation.

## Figures and Tables

**Table 1:** Baseline characteristics of all randomized patients.

	Fish oil *n* = 25	Placebo *n* = 25	*P* value
Gender, women, %	16.6%	17.7%	0.89
Age, years	35.1 ± 7.6	34.7 ± 7.8	0.85
BMI, Kg/m^2^	25.29 ± 3.9	24 ± 3.5	0.23
EDSS score	2.1 ± 0.98	2.06 ± 0.84	0.87
Evolution of disease, years	7.14 ± 4.79	6.68 ± 5.69	0.75
Total relapse before the study	5.44 ± 4.30	5.80 ± 6.93	0.82

Data are expressed in *n*, percentage (%) or mean and standard deviation (SD). Index of body mass (IBM), expanded disability status scale (EDSS), and Mann-Whitney *U* test.

**Table 2:** Effect of fish oil and placebo on cytokines, stress oxidative markers, and clinical outcomes.

	Baseline		*P* value	3 months		*P* value	6 months		*P* value	9 months		*P* value	12 months		*P* value
	Mean	SD		Mean	SD		Mean	SD		Mean	SD		Mean	SD	
TNF*α*, pg/mL															
Fish oil	39.8	4.7	0.66	37.4	3.9	0.01	30.8	3.1	<0.001	26.6	1.8	<0.001	22.7	2.4	<0.001
Placebo	39.4	5.6		39.5	2.6		40.2	1.5		39.9	1.1		39.1	3.1	
IL-1b, pg/mL															
Fish oil	44.6	4.2	0.46	40.9	3.8	0.27	28.8	1.6	<0.001	25.2	2.4	<0.001	22.2	1.8	<0.001
Placebo	44.1	4.3		40.5	2.5		40.0	1.4		40.0	1.5		37.4	8.1	
IL-6, pg/mL															
Fish oil	689.8	48.9	0.21	657.6	50.7	0.1	558.8	38.0	<0.001	407.6	48.7	<0.001	356.7	31.7	<0.001
Placebo	669.9	61.9		672.4	44.2		711.2	31.8		671.2	42.4		644.6	57.3	
NO, *μ*mol/L															
Fish oil	12.5	1.7	0.68	12.2	0.9	<0.001	9.4	0.8	<0.001	8.3	0.8	<0.001	8.0	0.9	<0.001
Placebo	12.8	1.5		13.7	0.7		14.2	0.7		13.9	0.7		13.7	0.8	
Lpo, *μ*mol/L															
Fish oil	2.5	0.98	0.64	3.1	0.90	0.96	2.9	1.1	0.25	2.6	0.8	0.25	2.1	0.9	0.79
Placebo	2.4	1.0		2.8	1.1		2.6	1.1		2.3	1.2		1.9	0.8	
EDSS, score															
Fish oil	2.1	0.98	0.66	—	—	—	2.1	0.9	0.73	—	—	—	2.2	1.0	0.66
Placebo	2.0	0.84		—	—		2.0	0.8		—	—		2.2	0.8	
Relapses rate, number															
Fish oil	—	—	—	—	—	—	—	—	—	—	—	—	0.84	0.9	0.79
Placebo	—	—		—	—		—	—		—	—		1	1	

Data expressed in mean and standard deviation (SD), Mann-Whitney *U* test.
